# Genome-Wide Association Study for Identification and Validation of Novel SNP Markers for *Sr6* Stem Rust Resistance Gene in Bread Wheat

**DOI:** 10.3389/fpls.2018.00380

**Published:** 2018-03-27

**Authors:** Amira M. I. Mourad, Ahmed Sallam, Vikas Belamkar, Stephen Wegulo, Robert Bowden, Yue Jin, Ezzat Mahdy, Bahy Bakheit, Atif A. El-Wafaa, Jesse Poland, Peter S. Baenziger

**Affiliations:** ^1^Department of Agronomy and Horticulture, University of Nebraska–Lincoln, Lincoln, NE, United States; ^2^Department of Agronomy, Faculty of Agriculture, Assiut University, Assiut, Egypt; ^3^Department of Genetics, Faculty of Agriculture, Assiut University, Assiut, Egypt; ^4^Department of Plant Pathology, University of Nebraska–Lincoln, Lincoln, NE, United States; ^5^United States Department of Agriculture-Agricultural Research Service, Manhattan, KS, United States; ^6^Cereal Disease Laboratory, United States Department of Agriculture-Agricultural Research Service, St. Paul, MN, United States; ^7^Plant Sciences Center, Department of Agronomy, Kansas State University, Manhattan, KS, United States

**Keywords:** genome-wide association study, linkage disequilibrium, marker-assisted selection, single marker analysis, SNP validation, haplotypes

## Abstract

Stem rust (caused by *Puccinia graminis* f. sp. *tritici* Erikss. & E. Henn.), is a major disease in wheat (*Triticum aestivium* L.). However, in recent years it occurs rarely in Nebraska due to weather and the effective selection and gene pyramiding of resistance genes. To understand the genetic basis of stem rust resistance in Nebraska winter wheat, we applied genome-wide association study (GWAS) on a set of 270 winter wheat genotypes (A-set). Genotyping was carried out using genotyping-by-sequencing and ∼35,000 high-quality SNPs were identified. The tested genotypes were evaluated for their resistance to the common stem rust race in Nebraska (QFCSC) in two replications. Marker-trait association identified 32 SNP markers, which were significantly (Bonferroni corrected *P <* 0.05) associated with the resistance on chromosome 2D. The chromosomal location of the significant SNPs (chromosome 2D) matched the location of *Sr6* gene which was expected in these genotypes based on pedigree information. A highly significant linkage disequilibrium (LD, *r^2^*) was found between the significant SNPs and the specific SSR marker for the *Sr6* gene (*Xcfd43*). This suggests the significant SNP markers are tagging *Sr6* gene. Out of the 32 significant SNPs, eight SNPs were in six genes that are annotated as being linked to disease resistance in the IWGSC RefSeq v1.0. The 32 significant SNP markers were located in nine haplotype blocks. All the 32 significant SNPs were validated in a set of 60 different genotypes (V-set) using single marker analysis. SNP markers identified in this study can be used in marker-assisted selection, genomic selection, and to develop KASP (Kompetitive Allele Specific PCR) marker for the *Sr6* gene.

**Highlights:**

Novel SNPs for Sr6 gene, an important stem rust resistant gene, were identified and validated in this study. These SNPs can be used to improve stem rust resistance in wheat.

## Introduction

Stem rust (caused by *Puccinia graminis* f. sp. *tritici* Erikss. & E. Henn.) is one of the most damaging diseases in wheat (Singh et al., 2006). It occurred frequently in the United States from the 1920s to 1960s and caused up to 50% yield losses (Leonard and Szabo, 2005). In the central United States Great Plains, an area from central Texas through central Nebraska, stem rust was a major disease and caused significant reductions in the wheat grain yield (Eversmeyer and Kramer, 2000). However, in recent years, stem rust occurs rarely at Nebraska, in part due to the effective selection for stem rust resistance by the USDA-ARS and University of Nebraska–Lincoln (UNL) Wheat Improvement Team for the past 60 years (Baenziger et al., 2001). The selection was phenotypically performed with little additional information on the genes existing in the germplasm and controlling the resistance except for pedigree information and the use of different pathotypes. Identifying genes controlling stem rust resistance by molecular markers is useful to accelerate breeding programs to improve stem rust resistance by identifying the genes, their vulnerabilities, and being able to pyramid multiple genes in wheat (Nisha et al., 2015).

With the advances in sequencing technologies, genotyping methods that combine variant discovery and genotyping in a single step are now being routinely used in plant breeding research. Genotyping-by-sequencing (GBS) is one such technique that involves the use of restriction enzymes for targeted complexity reduction of the genome followed by multiplexing and sequencing. Genotyping-by-sequencing can generate numerous SNP markers covering a high percentage of the genome in a cost-effective manner (Elshire et al., 2011; Poland and Rife, 2012). Therefore, these genome-wide SNPs can be used in genomic selection, genome-wide association study (GWAS) and genetic diversity studies.

Association mapping (AM) is a powerful approach which identifies polymorphisms near or within a gene of interest that controls the phenotypic differences between genotypes (Soto-Cerda and Cloutier, 2012). To perform AM, it is recommended that 100–500 individuals and codominant SSR or SNP markers are used (Kumar et al., 2011). Allele frequency distribution affects the power of AM to detect an association at the functional polymorphism level. Therefore, rare alleles, present in only a few individuals, cause a problem in association analysis due to their influence on the resolution power of mapping (Pearson and Manolio, 2008). In order to remove the effect of the rare alleles in AM, GBS derived SNPs can be filtered for at least 5% minor allele frequency (Soto-Cerda and Cloutier, 2012).

Few specific SNPs have been published or used for marker-assisted selection (MAS) for stem rust genes. Some exceptions are *Sr2* (Mago et al., 2011), *SrCad* (Kassa et al., 2016) and *Sr36*^1^ that have a SNP marker identified and used in MAS. No SNPs have been identified to be associated with some important stem rust resistance genes such as *Sr6, Sr30, Sr38*, and *Sr24*. Linkage disequilibrium (LD) analysis between specific SSR markers previously identified for stem rust resistance genes and SNPs generated from GBS with known chromosomal positions will be useful in identifying SNPs that are tightly associated with the stem rust gene of interest. Of course, these linkages between the detected SNPs and the target gene in diverse backgrounds will need to be confirmed.

The objectives of this study were to (1) screen a nursery of 270 Nebraska winter wheat genotypes for their resistance to stem rust race (QFCSC), the common race in the United States, (2) identify SNP markers associated with stem rust resistance using GWAS, (3) validate the SNPs associated with the resistance in another Nebraska winter wheat nursery, and (4) determine whether the markers identified in this study are located in genes and examine their potential role in disease resistance using functional annotations.

## Materials and Methods

### Plant Materials

To identify the SNP markers associated with stem rust resistance, a set of 270 winter wheat genotypes from the 2015 F_3:6_ nurseries (Nebraska Duplicate Nursery – DUP2015, the preliminary yield trial) were used. These genotypes were derived from 800 to 1000 crosses made among primarily Great Plains adapted genotypes with a heavy emphasis on using lines adapted specifically to Nebraska. These 270 genotypes were named as the association set (A-set). To validate the SNP markers associated with resistance in the A-set, a set of 60 genotypes from the 2015 F_3:7_ nurseries (Nebraska Triplicate Nursery-TRP2015, the advanced yield trial) were used and named the validation set (V-set). The TRP2015 nursery is derived from the selections from the DUP2014 nursery and do not overlap with the DUP2015. The V-set genotypes are selected and advanced from the DUP2014 nursery based on the grain yield, grain volume weight, disease resistance, plant height and maturity criteria (El-Basyoni et al., 2013). Three check cultivars were included in the evaluation: ‘Robidoux’ (moderately resistant to moderately susceptible to stem rust race QFCSC), ‘Freeman’ (moderately resistant to moderately susceptible to stem rust race QFCSC), and ‘Goodstreak’ (moderately resistant to stem rust race QFCSC).

### Stem Rust Inoculation and Screening

Both sets (A-set and V-set) were evaluated for stem rust resistance using the common stem rust race in Nebraska “QFCSC.” The A-set was evaluated for its resistance in two replications; one at the plant pathology greenhouses, University of Nebraska Lincoln, UNL and the other at USDA-ARS at Kansas State University (KSU). The V-set was evaluated for its resistance in two replications also, one at the plant pathology greenhouses, University of Nebraska Lincoln, UNL and the other at the USDA Cereal Disease Laboratory, St. Paul, MN, United States. The inoculation was performed at the seedling stage as described by Jin and Singh (2006) with minor modifications. Capsules of stored urediniospore were first removed from the freezer and thawed for 30 min at room temperature. Then, they were mixed with lightweight soltrol oil. Primary leaves of 10-day old seedlings were inoculated by atomizing a urediniospores suspension until the leaves were completely covered. Inoculated seedlings were placed in a transparent plastic mist chamber for 12 h in the dark at 20°C. Consequently, seedlings were placed on the greenhouse bench at 20 ± 2°C and a 16-h photoperiod for 2 weeks. The resistance was scored based on the scale of 0–4 as described by Stakman et al. (1962). Plants with an infection type (IT) score 0–2 were considered as resistant, and plants with IT scores 3–4 were considered as susceptible.

### Statistical Analysis of Stem Rust Resistance

Phenotypic data were converted from the Stakman et al. (1962) scale to a linear scale (L-IT) as mentioned in Kumssa et al. (2015) and Zhang et al. (2011) in order to convert the phenotypic data from a qualitative to a quantitative scale for analyses. The converted scores of stem rust resistance ranged from 0 (resistant) to 9 (susceptible). In the converted scale, scores of resistant genotypes ranged from 0 to 5 and those of susceptible genotypes ranged from 6 to 9. For complex IT range scores such as, “;, 1, 2”, only the lowest (“;”) and highest (“2”) IT scores were converted to the linear scale and then averaged. In both nurseries, the analysis of variance was performed with R software (R Core Team, 2017) using the following model

$$Y_{ij}=\mu +g_{i}+r_{j}+e_{ij}$$

where Y*_ij_* is an observation of genotype *i* in replication *j*, μ is the general mean; g*_i_* and r*_j_* are the main effects of genotypes (fixed effects) and replications (random effects), respectively; e*_ij_* is the error. The broad sense heritability (H^2^) was calculated using the following formula:

$$H^{2}=\sigma_{G}^{2}/\left(\sigma_{G}^{2}+\frac{\sigma_{R}^{2}}{r}\right)$$

where $\sigma_{G}^{2}$ and $\sigma_{R}^{2}$ are the variance of the lines and the residuals and r is the number of replicates within the experiment.

### DNA Extraction

DNA was extracted from lines in the A-set and V-set for GBS using BioSprint 96 DNA Plant Kits (Qiagen, Hombrechtikon, Switzerland) from 2 to 3 leaves of 2-weeks-old seedlings. For the SSR marker test, DNA from a bulk of six leaves of 5 days old plants was extracted using DNAzol Reagent (Molecular Research Center, Inc. Technical Bulletin 6). The tissue was ground using liquid nitrogen then 300 μl DNAzol reagent was added to this powder and left for 5 min at the room temperature. A volume of 300 μl chloroform was added to the previous mix and left at the room temperature for 5 min before it was centrifuged using Fisher Scientific accuSpin Micor 17 centrifuge (Loughborough, United Kingdom) at 12000 × g for 10 min. The washing process was done in three steps by adding three different washing solutions as follows: (1) absolute alcohol, (2) DNAzol + 75% Ethanol and (3) 75% alcohol only. All genotypes were centrifuged using the Fisher Scientific accuSpin Micor 17 centrifuge for 4 min at 5000 × g after each washing step. The extracted DNA was then re-suspended in 150 μl of TE buffer. DNA concentration was measured using spectrophotometry (Gen5^TM^ microplate reader and image software with Take3^TM^ micro-volume plates [BioTek, Winooski, VT, United States]).

### Genotype-by-Sequencing (GBS)

The DNA of both sets (A-set and V-set) were genotyped using GBS by digesting the DNA with two restriction enzymes, *PstI* and *MspI* (Poland and Rife, 2012). Pooled libraries were sequenced using Illumina, Inc. NGS platforms. The FASTQ file containing the raw data of sequence reads were processed for SNP identification using TASSEL 5.0 v2 GBS pipeline (Bradbury et al., 2007). Chinese Spring genome assembly from the International Wheat Genome Sequencing Consortium (IWGSC) Reference Sequence v1.0 was used as the reference genome. Raw sequence data of the tested genotypes along with additional ∼3000 breeding lines from the University of Nebraska wheat program were combined to increase the genome coverage and read depth for SNP discovery in both nurseries. SNP markers identified were filtered for minor allele frequency (MAF > 0.05), maximum missing sites per SNP < 20% and maximum missing sites per genotype < 20% (Belamkar et al., 2016). Heterozygous loci were then marked as missing to obtain better estimates of marker effects (Peter Bradbury, personal communication) and the SNP markers were re-filtered using the same filtering criteria.

### Population Structure

The analysis of population structure was performed on the A-set using the Baysian model-based software program STRUCTURE 3.4 (Pritchard et al., 2000). For each run, burn-in iteration was 100,000, followed by 100,000 Markov chain Monte Carlo (MCMC) replications after burn-in. The admixture and allele frequencies correlated models were considered in the analysis. Five impended iterations were used in the STRUCTURE. The hypothetical number of the subpopulation (k) extended from 1 to 10. The best k was identified according to Evanno et al. (2005) using STRUCTURE HARVESTER (Earl and vonHoldt, 2012).

### Genome-Wide Association Study (GWAS)

Stem rust resistance data of the A-set were used to identify SNPs associated with the resistance. The association of the SNP markers retained after quality-filtering and stem rust measurements was carried out using TASSEL 5.0 software (Bradbury et al., 2007) using a mixed linear model (MLM: Yu et al., 2006). The marker-trait association was tested against Bonferroni corrections at a significance level of 5%. The allele estimates of each marker determine the influence of the allele on the phenotype. In the linear scale used for phenotypic measurements, smaller values represent resistance and larger values indicate susceptibility. Hence, alleles with a lower marker effect score are effective alleles and are linked to increasing resistance in the population. The phenotypic variation explained by a marker (*R*^2^) was calculated for the significant SNPs using TASSEL 5.0 (Bradbury et al., 2007). The Q–Q and Manhattan plots of the GWAS were developed using ‘qqman’ R package (Turner, unpublished).

For the significant SNP markers detected by GWAS, the alleles associated with increased stem rust resistance were assigned a value of 1 while those associated with decreased stem resistance were marked 0 to perform a correlation analysis with the specific *Sr6* SSR marker. Linkage disequilibrium (*r^2^*) among the significant SNPs and the specific SSR marker located on the same chromosome was calculated by TASSEL 5.0 and visualized as a heatmap using ‘LDheatmap’ R package (Shin et al., 2006).

### Haplotype Block Analysis

Haplotype block analysis was performed using Haploview 4.2 software (Barrett et al., 2005). The SNP data of the A-set was used to calculate the pair-wise LD between SNPs. A cutoff of 1% was used, meaning that if the addition of a SNP to a block resulted in a recombinant allele at a frequency exceeding 1%, then that SNP was not included in the block. The Haplotype blocks were constructed using the four-gamete methods which create block boundaries where there is evidence of recombination between adjacent SNPs based on the presence of all four gametic types (Wang et al., 2002).

### Polymerase Chain Reaction (PCR) Conditions

Polymerase chain reaction (PCR) for the available SSR primers of the *Sr6* gene (*Xcfd43* and *Xwmc453*) was performed in 15 μl volume with 2 μl 20 ng DNA template, 3 μl GoTaq flexi buffer (without MgCl_2_), 0.3 μl 0.25 mM dNTPs, 1.20 μl 25 mM MgCl2, 0.2 μl from 0.5 μ/μl GoTaq Flexi Taq Polymerase (Promega, Madison, Wisconsin, USA) and 0.75 μl 10 pmol from each primer. SSR marker products were separated in super fine resolution (SFR) 3% Agarose gel. The differential line for the *Sr6* gene (ISr6-Ra CI 14163) was included in the SSR test to identify the target band size for each primer. The SSR products gel was scored visually and by using Gel Analyzer 2010 software.^2^

### Single Marker Analysis (SMA) for Marker Validation

Phenotypic data of the V-set, using the converted linear scale (Kumssa et al., 2015), as well as genotypic data of markers which were associated with stem rust in the A-set were used to perform single marker analysis. The analysis was done by using SAS Version 9 (SAS Institute Inc., 2013) following this model:

Y =μ+f(mar⁢ker⁡)+error,

where Y is equal to the trait value, μ is equal to the population mean, and f (marker) is a function of the significant markers (Francis et al., 2011).

### Genes Containing Significant SNPs and Their Annotations

To further investigate the GWAS results, we inspected whether any of the significant SNPs were in genes identified and annotated in the reference genome assembly (IWGSC Ref Seq v1.0). The effect of the significant SNPs on annotated genes was determined by using SnpEff (Cingolani et al., 2012). Functional annotation of the genes harboring significant SNPs were retrieved from the genome annotations provided by IWGSC and examined for their association with stem disease resistance.

## Results and Discussion

**FIGURE 1:**
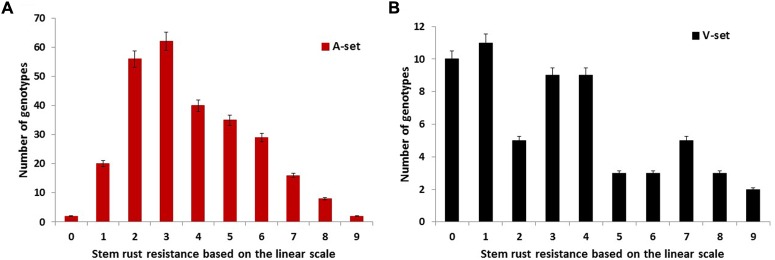
The infection response of the genotypes in the **(A)** association set (A-set, 270 genotypes) and **(B)** validation set (V-set, 60 genotypes) to infection with the common stem rust race in Nebraska, QFCSC using the linear scale of Kumssa et al. (2015).

The analysis of variance revealed highly significant differences among genotypes (**Table 1**), indicating our phenotypic assay was successful. Such a high genetic variation found among genotypes could be very useful for plant breeders to select the most resistant genotypes to stem rust. **Figure 1A** represents the response of the A-set to the most common Nebraska stem rust race (QFCSC). The A-set was mostly resistant (80% of the genotypes) with L-IT ranging from 0 to 5. This result was expected because the previous generations were screened for stem rust resistance and the selection was made using stem rust resistance as one of the selection criteria.

**Table 1:** Analysis of variance for stem rust resistance in the association set (A-set) of 270 genotypes and the validation set (V-set) of 60 genotypes.

	A-set		V-set	
		
Source	D.F.	M.S.	D.F.	M.S.
Replications	1	20.15^∗^	1	23.3
Genotypes	269	6.75^∗∗^	59	13.40^∗∗^
Error	263	4.08	58	6.07
Total	533		118	
Broad-sense heritability (%)	39.59		54.68	


*^∗^*p <* 0.05, ^∗∗^*p <* 0.01.*

### Association Mapping for Stem Rust Resistance

#### Population Structure

The GBS generated a set of 35,216 SNPs after filtering for minor allele frequency (MAF > 0.05), maximum missing sites per SNP < 20% and maximum missing sites per genotype < 20% (Belamkar et al., 2016) (Data are available on doi: 10.6084/m9.figshare.5948416). This set of SNPs was used in population structure analysis. After marking the heterozygous loci as missing values and re-filtering the data, 23,053 SNPs and a set of 259 genotypes in the A-set nursery and 60 genotypes in the V-set were used in our marker-trait association studies. The 23,051 SNPs were used in the GWAS analysis.

**FIGURE 2:**
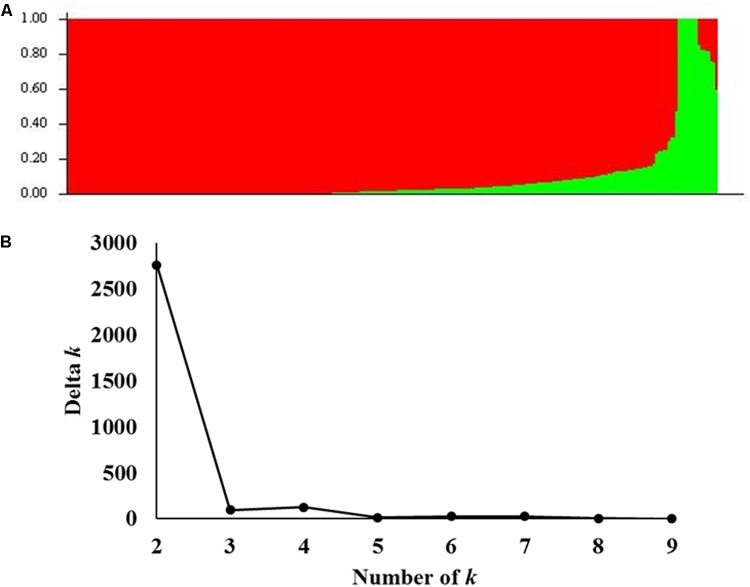
Analysis of population structure using 35,216 SNP markers: **(A)** Estimated population structure of 259 winter wheat genotypes (*k* = 2). The *y*-axis is the sub-population membership, and the *x*-axis is the genotypes. **(B)** Delta-k for different numbers of sub-populations as determined by Evanno et al. (2005).

Population structure analysis was performed on the A-set and two possible subpopulations were identified (**Figure 2A**). To confirm this result, the number of suggested k was plotted against the calculated Δk obtained from STRUCTURE software. A clear peak was observed for *k* = 2 (**Figure 2B**). Therefore, a *k* value of two was chosen to describe the genetic structure of the genotypes used in this study. That the structure was found in the A-set was expected due to the selection process for important agronomic traits and different climatic zones in the Nebraska wheat breeding program.

#### Genome-Wide Association Study and Linkage Disequilibrium

**FIGURE 3:**
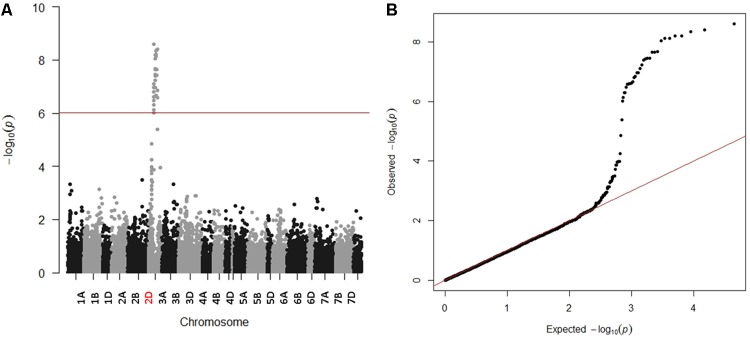
**(A)** Manhattan plot displaying SNP marker-trait association identified for stem rust resistance in GWAS using 259 winter wheat lines of the A-set. Redline is significance threshold of 5% Bonferroni correction. **(B)** Quantile-Quantile (QQ) plot used to evaluate the performance of the mixed linear model used for of GWAS for stem rust resistance using mixed linear model (MLM).

**FIGURE 4:**
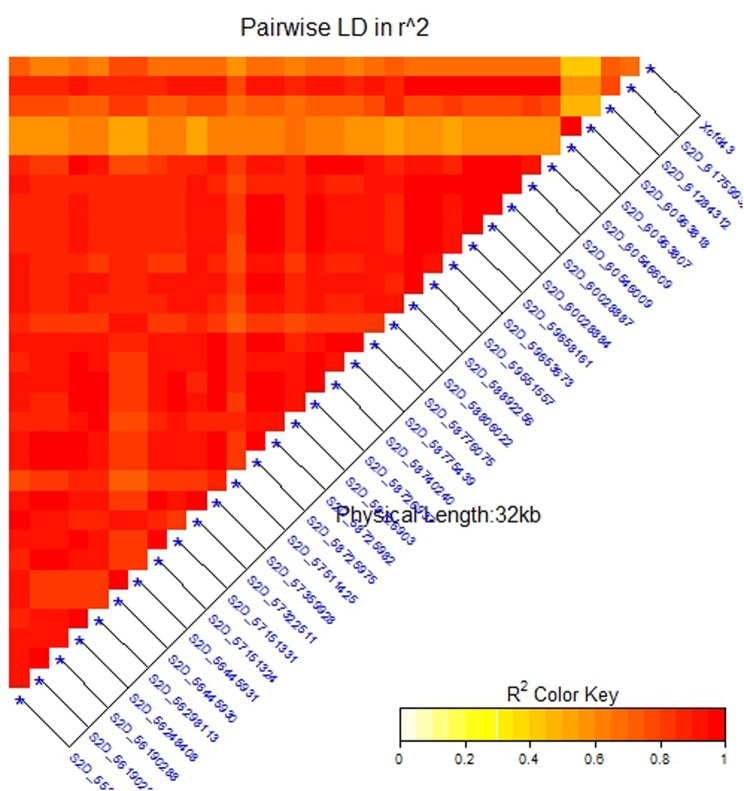
Linkage disequilibrium (LD) analysis in the association set (A-set): heatmap of LD between the significant SNPs detected by the mixed linear model (MLM) (at a significance level of 5% Bonferroni correction) and SSR marker (*Xcfd43*) that predicts *Sr6.*

Due to the presence of structure in our tested population, MLM was used for GWAS. Association analysis identified 32 significant SNPs for stem rust resistance based on Bonferroni correction (α = 0.05) (**Table 2** and **Figures 3A,B**). Remarkably, all the significant SNPs were located on chromosome 2D. The phenotypic variation explained by each SNP marker (R^2^) ranged from 11.70 to 17.68%. The *R*^2^ for all significant SNPs indicates that these SNPs represented a major QTL associated with stem rust resistance. Allele C of SNP marker S2D_57151324 had the lowest allele effect which is associated with decreasing the symptoms of stem rust (-1.48), while allele C in S2D_61759932 had the highest allele effect (-1.86) associated with decreased the symptoms of stem rust. Among all the 32 SNPs, S2D_61759932 showed the highest allele effects (-1.86) with *R*^2^ of 16.75%. The pair-wise LD between the 32 SNPs is illustrated in **Figure 4**. Highly significant LD was found among these 32 significant SNPs identified based on Bonferroni correction. This result indicated that the 32 SNPs may tag the same QTL and seem to be co-inherited together.

**Table 2:** Association analysis of stem rust resistance using the mixed linear model (MLM) and the correlation between the significant SNPs and *Xcfd43* markers which tags *Sr6*.

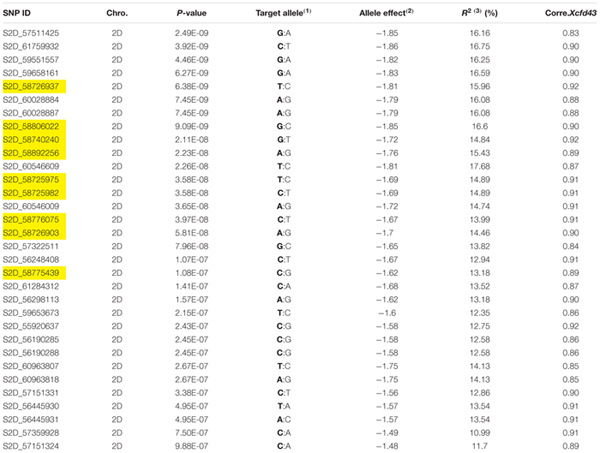

*^(*1*)^*The allele on the left increased the resistance*. ^(*2*)^The effect of left allele associated with increased resistance. ^(*3*)^Phenotypic variation explained by marker. Highlighted SNPs locate in haplotype block 81. In each SNP_ID, S2D referrers to chromosome 2D followed by the position of that SNP on the chromosome.*

Several previously identified stem rust resistance genes such as *Sr6*, *Sr46* and *Sr32* are mapped to chromosome 2D.^3^ However, based on the available pedigree information of the tested populations, we expected to have the *Sr6* gene and not to have *Sr32* and *Sr46* genes in our materials. To confirm the presence of the *Sr6* gene in the tested population, specific SSR markers (*Xcfd43* and *Xwmc453*) were used in genotyping the genotypes. Only the *Xcfd43* marker presented clear amplification, while *Xwmc453* did not give good amplification. The result of the *Xcfd43* marker revealed that of the 270 genotypes, 123 (47%) were found to be containing the target allele associated with the *Sr6* gene. The correlation between the specific SSR marker and each one of the significant SNPs is presented in **Table 2**. The 32 significant SNPs revealed high correlations with the specific SSR marker with *r*^2^ values ranging from 0.83 for marker S2D_57511425 to 0.92 for markers S2D_58726937, S2D_58740240 and S2D_55920637. This result indicates that these SNPs were tightly linked to *Sr6*. A High LD was found between the SSR marker (*Xcfd43*) and all the significant SNPs (**Figure 4**). This high LD between the specific SSR marker for the *Sr6* gene and the significant SNPs suggested (1) the SSR marker is highly co-inherited with the 32 SNPs, and (2) it is considered as a further support that some of these significant SNPs could be a part of this gene. Such high LD is very useful for marker-assisted selection. The significant SNPs were removed from the marker data and the AM was re-analyzed to find out if the LD among the 32 markers interferes with the effect of other SNPs with small effects and associated with stem rust resistance. No significant markers were associated with stem rust resistance. This result is additional evidence of the power of GWAS performed in this study.

To confirm that the significant 32 SNPs are identifying the *Sr6* gene, we inspected the location of the *Sr6* SSR primer using the IWGSC RefSeq v1.0 marker mapping file^4^. The mapping file released by the IWGSC includes location information for previously mapped SSR markers. The sequence of the SSR marker (*Xcdf43*) has been mapped to a scaffold that is currently not included in any of the chromosomes and is placed along with other scaffolds that lack location information in an “Unknown” chromosome in the IWGSC RefSeq v1. Due to the high LD between the *Sr6* SSR marker and the significant SNPs as well as the previous study that has placed the SSR marker for *Sr6* on chromosome 2D (Tsilo et al., 2010), we believe the scaffold containing the *Sr6* sequence can be placed on chromosome 2D.

#### Genes Underlying Significant SNPs and Their Functional Annotations

To further understand the association between the significant SNPs and the stem rust resistance, we reviewed the annotation of genes containing these SNPs and studied the effect of the SNPs on the genes. Out of the 32 SNPs, eight SNPs are located within genes: TraesCS2D01G108000.1 (two SNPs), TraesCS2D01G104700.1 (two SNPs), TraesCS2D01G104400.1 (one SNP), TraesCS2D01G104600.1 (one SNP), TraesCS2D01G106100.1 (one SNP) and TraesCS2D01G107200.1 (one SNP). Four of these eight SNPs result in an amino acid change (missense variant), one of the SNPs causes the loss of stop codon, and the rest of the three are synonymous SNPs (Supplementary Table 1). The SNP in the gene, TraesCS2D01G106100.1, results in loss of stop codon and is the highest impact variant. The loss of stop codon results in degradation of the transcript in the nucleus, and thus a complete loss of function of the gene (Fasken and Corbett, 2005). The functional annotation of some of the genes indicates their potential involvement in fungal disease resistance (**Table 3**). For example, TraesCS2D01G104700.1 and TraesCS2D01G104600.1 are WRKY transcription factors and the role of the WRKY transcription factor family is well-known in biotic stresses including disease resistance (Pandey and Somssich, 2009; Wang et al., 2013; Phukan et al., 2016). The TraesCS2D01G104400.1 is an E3-ubiquitin-protein ligase and these genes have been shown to contribute to resistance to fungal diseases such as powdery mildew (caused by *Blumeria graminis* f. Sp. *tritici*) (Zhu et al., 2015). The F-box domain containing protein (TraesCS2D01G106100.1) was found to have an effect on disease resistance in tomato *(Solanum lycopersicum* L.) and tobacco (*Nicotiana tabacum* L.) (Maclean et al., 2008). In summary, many of the genes underlying significant SNPs have been annotated as being involved in disease resistance which further validated the marker-trait associations identified in this study.

**Table 3:** Gene models underlying significant SNPs and their annotations from the International Wheat Genome Sequencing Consortium reference genome assembly v1.0 of the variety Chinese spring.

SNP ID	Gene model	Gene annotation	Probable function
S2D_60028884	TraesCS2D01G108000.1	Heat shock 70 kDa protein	Heat stress resistance
S2D_60028887			
S2D_56445930	TraesCS2D01G104700.1	WRKY transcription factorPF0310	Abiotic and biotic stresses resistance
S2D_56445931			
S2D_56248408	TraesCS2D01G104400.1	E3-ubiquitin-protein ligase	
S2D_56298113	TraesCS2D01G104600.1	WRKY transcription factorPF0310	Abiotic and biotic stresses resistance
S2D_57359928	TraesCS2D01G106100.1	F-box domain containing protein	
S2D_59551557	TraesCS2D01G107200.1	WEAK movement UNDER BLUE LIGHT-like protein	Controlling Chloroplasts accumulate


#### Haplotype Block Analysis

**FIGURE 5:**
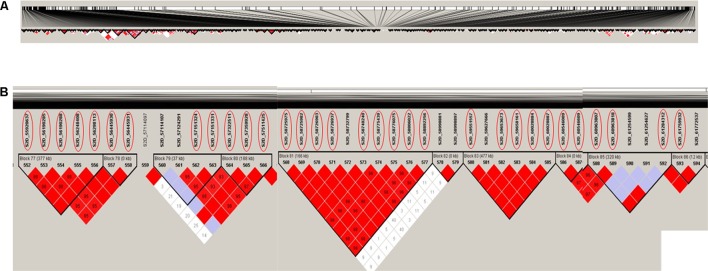
Haplotype block analysis for chromosome 2D, **(A)** the 306 haplotype blocks on the whole chromosome, **(B)** the nine haplotype blocks containing the 32 significant SNPs associated with stem rust resistance. SNPs with red circles are significantly associated with stem rust resistance based on 5% Bonferroni correction.

Haplotype block analysis revealed the existence of 306 haplotype blocks on chromosome 2D (**Figure 5A**). The 32 significant SNPs were located within nine haplotype blocks with numbers 77, 78, 79, 80, 81, 83, 84, 85, and 86 (**Figure 5B**). Block 81 contains most of the significant SNPs (nine SNPs), while the remaining markers were distributed in eight different blocks (**Figure 5B**). All the 32 significant SNPs are located on haplotype blocks adjacent to each other (on block number 77 through 86). This result indicated that these SNPs most likely identify the same QTL. The haplotype blocks shed light on genomic regions associated with the trait following a GWAS study. For example, selecting the haplotype block enriched with significant SNPs as compared to using individual SNPs can reduce redundancy introduced by using all SNPs (Mokry et al., 2014). Based on the GWAS and haplotype block analysis, we recommend the significant SNPs located in block 81 (**Table 2**) as the best targets for marker-assisted selection to improve stem rust resistance in seedling winter wheat.

#### Validation of the SNPs in the Validation Set (V-set)

To validate the association between the significant SNPs and stem rust resistance, a set of 60 genotypes was evaluated for QFCSC stem rust race in two replications. Highly significant differences were found between the genotypes with no significant differences between the replications (**Table 1**). Seventy-eight percent of the genotypes were resistant with L-IT scores ranging from 0 to 5 which indicated the possibility of these genotypes to be used for the validation purpose (**Figure 1B**).

Based on single marker analysis, all the significant SNPs identified in the A-set had a strong significant association with stem rust resistance in the V-set (**Table 4**). The *F-values* of the significant SNPs ranged from 8.91 to 30.96. The *R*^2^ for the significant SNPs ranged from 15.09 to 38.72% which indicates that these SNPs explain the high percentage of the phenotypic variation in the V-set.

**Table 4:** Single marker analysis (SMA) for the 32 significant SNPs from the association set (A-set) and the *Xcfd43* marker which predicts the presence of *Sr6* in the validation set (V-set).

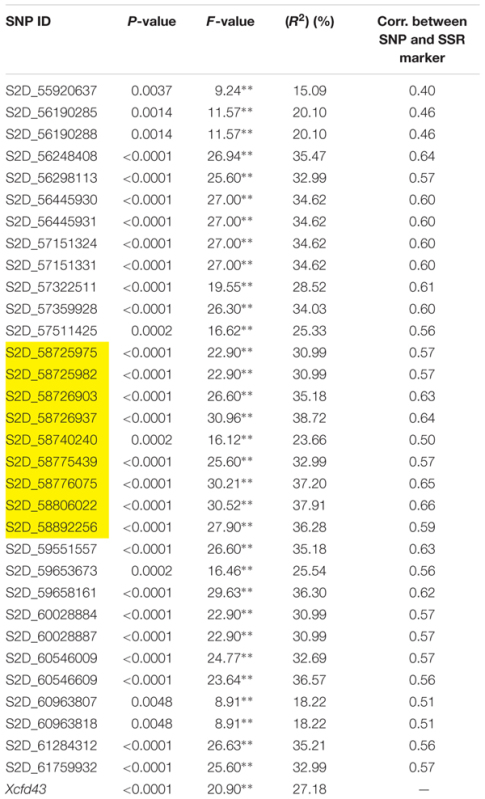

**R*^*2*^ the phenotypic variation explained by the marker. ^∗∗^Highly significant association between the SNP marker and the stem rust resistance in the V-set.*

**FIGURE 6:**
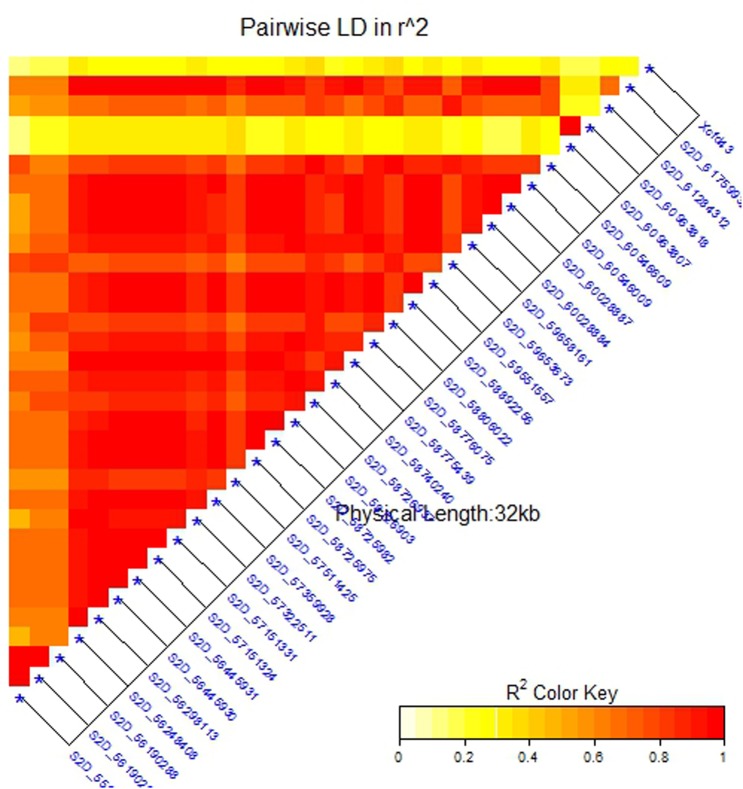
Linkage disequilibrium (LD) analysis in the validation set (V-set): heatmap of LD between the significant SNPs detected by the mixed linear model (MLM) (at significance level of 5% Bonferroni correction) and SSR marker *Xcfd43* that predicts *Sr6.*

**FIGURE 7:**
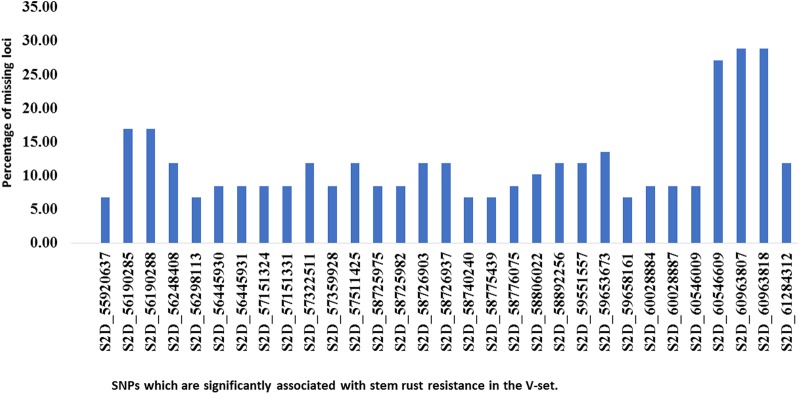
Histogram of the percentage of missing loci for each significant SNP (identified in the association set) in the validation set.

To confirm that the 32 SNPs were good markers for the *Sr6* gene, the V-set was genotyped using specific SSR marker for the *Sr6* gene (*Xcfd43*). Thirty–seven percent (22 genotypes) had the target band associated with *Sr6*. The correlation between the SSR marker and the significant SNPs (**Table 4**) ranged from 0.40 to 0.64. The LD between the SSR and the significant SNPs (**Figure 6**) was low. This result was explained by the high percentage of the missing SNPs in the V-set with a range extending from 6.78 to 28.81% (**Figure 7**). The high percentage of missing loci reduced the correlation and LD values. The significant association between the 32 SNPs and stem rust resistance in the V-set is evidence that these SNPs are very useful for a future breeding program to improve stem rust resistance in winter wheat where Ug99 pathotypes are not present. Validation of QTLs associated with target traits is one of the main steps of maker-assisted selection. The advantage of the V-set is that it represents a different genetic background from that used for GWAS (A-set). Generally, the 32 SNPs associated with stem rust resistance especially those that are located in block 81 (nine SNPs) could be converted to Kompetitive Allele Specific PCR (KASP) for the *Sr6* gene.

## Conclusion

In conclusion, our study for the first time identified 32 novel SNPs associated with *Sr6* which is an important gene providing resistance to a wide range of stem rust races (MCCFC, QCCSM, QFCSC, RCRSC, GFMNC, and TPMKC). The SNPs associated with stem rust in this study can be considered for MAS. However, the nine SNPs located within the same haplotype block and explain relatively high phenotypic variation are probably most promising. The reasons supporting this conclusion are (I) the high LD and correlation found between these SNPs and the *Xcfd43* marker (predicts the *Sr6* gene) and (II) the validation of the association between these SNPs and the *Sr6* gene with highly significant *p* and *R*^2^ values in a set of lines with different genetic backgrounds. These novel SNPs should be very useful for improving stem rust resistance.

## Author Contributions

AM performed all the genetic and phenotyping analyses and wrote the manuscript. AS helped in genetic and phenotypic analyses. VB performed the SNP calling from GBS data and helped in the genetic analysis. SW, RB, and YJ helped in phenotyping stem rust resistance. EM, AE-W, and BB helped in design the study. JP performed GBS of the two populations. PB designed the study, helped in discussing the results and drafted the manuscript. All the authors agreed to be accountable for the content of the work.

## Conflict of Interest Statement

The authors declare that the research was conducted in the absence of any commercial or financial relationships that could be construed as a potential conflict of interest.

## Footnotes

1 http://maswheat.ucdavis.edu/protocols/sr36/

2 http://www.gelanalyzer.com/

3 http://maswheat.ucdavis.edu/protocols/StemRust/StemRustMap.html

4 https://wheat.pw.usda.gov/cgi-bin/graingenes/report.cgi?class=probe;name=CFD43
