# Supplementary material for: Genome-Wide Association Study for Identification and Validation of Novel SNP Markers for Sr6 Stem Rust Resistance Gene in Bread Wheat
Source: Front Plant Sci. 2018 Mar 27;9:380. doi: 10.3389/fpls.2018.00380 (PMC5881291; doi:10.3389/fpls.2018.00380)
Supplement: Supplementary file 1 [file Table_1.DOCX]

Supplementary Table 1: Annotation and effect prediction of SNP markers associated with stem rust resistance

| **SNPID** | **Annotation** | **Annotation_Impact** | **Gene_Name** |
| --- | --- | --- | --- |
| S2D_55920637 | intergenic_region^1^ | Modifier | TraesCS2D01G103400-TraesCS2D01G103500 |
| S2D_56190285 | upstream_gene_variant^2^ | Modifier | TraesCS2D01G104100 |
| S2D_56190288 | upstream_gene_variant | Modifier | TraesCS2D01G104100 |
| S2D_56248408 | synonymous_variant^3^ | Low | TraesCS2D01G104400 |
| S2D_56298113 | synonymous_variant | Low | TraesCS2D01G104600 |
| S2D_56445930 | missense_variant^4^ | Moderate | TraesCS2D01G104700 |
| S2D_56445931 | missense_variant | Moderate | TraesCS2D01G104700 |
| S2D_57151324 | upstream_gene_variant | Modifier | TraesCS2D01G105600 |
| S2D_57151331 | upstream_gene_variant | Modifier | TraesCS2D01G105600 |
| S2D_57322511 | intergenic_region | Modifier | TraesCS2D01G106000-TraesCS2D01G106100 |
| S2D_57359928 | stop_lost^5^ | High | TraesCS2D01G106100 |
| S2D_57511425 | intergenic_region | Modifier | TraesCS2D01G106100-TraesCS2D01G106200 |
| S2D_58725975 | intergenic_region | Modifier | TraesCS2D01G106400-TraesCS2D01G106500 |
| S2D_58725982 | intergenic_region | Modifier | TraesCS2D01G106400-TraesCS2D01G106500 |
| S2D_58726903 | upstream_gene_variant | Modifier | TraesCS2D01G106500 |
| S2D_58726937 | upstream_gene_variant | Modifier | TraesCS2D01G106500 |
| S2D_58740240 | intergenic_region | Modifier | TraesCS2D01G106500-TraesCS2D01G106600 |
| S2D_58775439 | upstream_gene_variant | Modifier | TraesCS2D01G106600 |
| S2D_58776075 | upstream_gene_variant | Modifier | TraesCS2D01G106600 |
| S2D_58806022 | downstream_gene_variant^6^ | Modifier | TraesCS2D01G106700 |
| S2D_58892256 | intergenic_region | Modifier | TraesCS2D01G106700-TraesCS2D01G106800 |
| S2D_59551557 | synonymous_variant | Low | TraesCS2D01G107200 |
| S2D_59653673 | upstream_gene_variant | Modifier | TraesCS2D01G107400 |
| S2D_59658161 | splice_region_variant^7^&intron_variant^8^ | Low | TraesCS2D01G107400 |
| S2D_60028884 | missense_variant | Moderate | TraesCS2D01G108000 |
| S2D_60028887 | missense_variant | Moderate | TraesCS2D01G108000 |
| S2D_60546009 | intergenic_region | Modifier | TraesCS2D01G108900-TraesCS2D01G109000 |
| S2D_60546609 | intergenic_region | Modifier | TraesCS2D01G108900-TraesCS2D01G109000 |
| S2D_60963807 | intergenic_region | Modifier | TraesCS2D01G109900-TraesCS2D01G110000 |
| S2D_60963818 | intergenic_region | Modifier | TraesCS2D01G109900-TraesCS2D01G110000 |
| S2D_61284312 | intergenic_region | Modifier | TraesCS2D01G110400-TraesCS2D01G110500 |
| S2D_61759932 | intergenic_region | Modifier | TraesCS2D01G110800-TraesCS2D01G110900 |

^1^ intergenic_region The SNP is in an intergenic region

^2^ upstream_gene_variant The SNP is in the upstream of a gene (default length: 5K bases)

^3^ synonymous_variant SNP causes a codon that produces the same amino acid

^4^ missense_variant SNP causes a codon that produces a different amino acid

^5^ stop_lost SNP causes stop codon to be mutated into a non-stop codon

^6^ downstream_gene_variant The SNP is in the downstream of a gene (default length: 5K bases)

^7^ splice_region_variant A sequence variant in which a change has occurred within the region of the splice site, either within 1-3 bases of the exon or 3-8 bases of the intron.

^8^ intron_variant SNP hits an intron
